# The Role of Behavioral Management in Enhancing Clinical Care and Efficiency, Minimizing Social Disruption, and Promoting Welfare in Captive Primates

**DOI:** 10.3390/vetsci11090401

**Published:** 2024-09-01

**Authors:** Scott H. Oppler, Sierra D. Palmer, Sydney N. Phu, Melanie L. Graham

**Affiliations:** 1 Department of Surgery, University of Minnesota, Minneapolis, MN 55455, USA; 2 Department of Veterinary Population Medicine, University of Minnesota, St. Paul, MN 55108, USA

**Keywords:** nonhuman primate, behavioral management, welfare, training, clinical care

## Abstract

**Simple Summary:**

Medical procedures can be stressful for both humans and animals alike. Stress and anxiety related to medical care can have negative effects on patient experience, quality of life, and, ultimately, health outcomes. In situations where there are no alternatives, nonhuman primates are used in biomedical research to study disease to develop lifesaving therapies. Captive management and routine healthcare necessitate that animals undergo potentially stressful medical procedures, such as physical examination, drug or fluid administration, blood collection, and other essential health management protocols. Implementing behavioral management strategies including enrichment, social interaction, and cooperative training fosters positive coping with these stressors, empowering them to actively participate in their own care. This can reduce stress and anxiety during required procedures, making medical care more efficient and less invasive, while also allowing veterinarians to perform examinations and administer treatments without compromise to the benefit of overall animal health and welfare, as well as improved clinical outcomes. In this study, we explore the broad impact of behavioral management on the experience of primates undergoing routine veterinary care, emphasizing its contributions in improving animal welfare, scientific rigor, and efficiency in care and monitoring practices.

**Abstract:**

Medical procedures necessary for routine care can induce stress in both the veterinary and human clinical situations. In the research environment, nonhuman primates undergo procedures like physical examination, blood sampling, and intravenous drug or fluid administration either as a part of routine veterinary care or during the modeling of clinical disease and interventions under study. Behavioral management techniques, such as training for cooperation, allow caregivers to train primates to voluntarily engage in various medical procedures. This approach reduces stress and anxiety associated with necessary procedures, thereby enhancing efficiency and minimizing the invasiveness of medical care. Consequently, veterinary evaluation and care can be provided without compromise, resulting in enhanced clinical outcomes and overall better health. In this study, we explored the impact of the behavioral management program implemented at our center on a subset of animals undergoing routine veterinary care, focusing on the overall experience, including animal welfare, scientific rigor, and efficiency in terms of economics and time. We investigated its impact on key factors, such as the total procedure and recovery time, incidence of side effects, and welfare indicators, revealing a significant positive influence on animal care. Furthermore, through case studies, we illustrate how behavioral management facilitates timely medical care and monitoring, effectively mitigating stressors that could otherwise impair health and welfare, enabling the provision of care that would have otherwise been unachievable. A thoughtfully designed primate behavioral management program, integrating cooperation and participation with veterinary care, forms the cornerstone of superior animal welfare, enhanced clinical care, and more accurate scientific outcomes.

## 1. Introduction

For both humans and animals alike, the experience of even routine medical procedures has the potential to induce stress and fear that can be detrimental to overall welfare, reduce patient satisfaction, and negatively impact effective patient care and health outcomes [1,2]. Anxiety related to experiences in the healthcare setting can even affect future medical decisions and impair the important trust and relationship between patient and caregiver [3]. This problem is becoming increasingly recognized in various healthcare settings, with strategies being developed to reduce stress and improve the patient experience. In pediatric medicine, examples include the implementation of integrated care with child life specialists trained to provide supportive interventions to enhance the patient experience [4,5], while in the veterinary setting, increased emphasis has been placed on the “Fear Free” concept, which comprises numerous refinements related to areas ranging from the clinic environment to handling and restraint methods aimed at reducing clinic-associated stress and fear [6,7,8]. The use of behavioral techniques including preparation and distraction has been successful in the pediatric setting at reducing self-reported fear and the total procedure time [5]. Ultimately, these strategies attempt to increase a patient’s ability to cope with the challenges of the medical environment, with the aim of improving patient quality of life and facilitating easier medical management.

Nonhuman primates (NHPs) continue to play a critical role in the research setting, where, in the absence of suitable alternatives, they continue to serve as a critical preclinical model for the evaluation of novel drugs and therapeutics owing to their physiologic and anatomic similarities to humans that allow them to uniquely represent the clinical situation [9,10,11]. In this role, NHPs are often exposed to potentially stressful, aversive medical procedures similar to those experienced by clinical patients, including physical examination, frequent handling for sampling, drug administration, and health management. Chronic and repeated stress from a range of sources not only affects welfare [12,13,14,15,16,17] but can have a significant impact on long-term health and responses to therapy [18], and, in scientific studies, unduly influence a range of outcome measures [19,20,21]. Further, animal fear and anxiety can complicate veterinary care and accurate monitoring by making it difficult to perform a thorough, comprehensive assessment of the animal. 

In the captive setting, the introduction of behavioral management techniques that combine enrichment, sociality, and training have the potential to improve animals’ ability to cope with these stressors [13,22]. Behavioral management techniques address the psychological needs of animals by implementing opportunities that encourage species-typical behaviors, reduce stress, and decrease abnormal behaviors, with the goal of improving animal welfare [13]. 

Cooperative training gives animals the opportunity to voluntarily participate in their own management and care and express agency in how they engage in, rather than experience, certain procedures under the more unpredictable, stress-inducing physical restraint or sedation that typically requires the temporary disruption of an animal’s social environment and may negatively impact the animal–caregiver bond, further impacting its long-term care and welfare [23]. Implementing a training paradigm designed to shape behavior by strategically adding or removing reinforcing stimuli in a manner that aligns with the animals’ preferences has demonstrated the ability to enable all animals, irrespective of age, sex, or temperament, to develop important positive coping skills [24]. Training for cooperation enhances safety during animal–caregiver interactions, benefitting both the animals and their handlers [20,25]. Moreover, it enables improved medical management by facilitating the application of therapies that might otherwise be challenging, such as repeated sampling or intravenous access for drug administration in animals where sedation is not advisable. 

While various behavioral management techniques are becoming more prevalent in research settings, few studies have explored their impact on the animal experience during routine veterinary procedures, which are necessary yet potentially aversive. In this study, we aimed to assess the influence of training for cooperation [24,26] on the overall experience of NHPs undergoing intravenous access for blood collection, a standard medical procedure essential for gathering diagnostic health information. We examined the duration of the procedure, occurrence of side effects, and impact on welfare factors, such as separation time from social partners. In this study, we explore the practical implications of a deliberate behavioral management program that incorporates cooperative training. We present case studies demonstrating how this approach enhances the execution of timely medical care and comprehensive health monitoring while minimizing the need for stress-inducing techniques like sedation. These examples underscore the critical relationship between behavioral management and high-quality veterinary care, highlighting how their integration serves as a cornerstone for superior animal welfare, improved clinical outcomes, and enhanced scientific rigor.

## 2. Materials and Methods

### 2.1. Animal Subjects

All animal procedures were approved by the University of Minnesota Institutional Animal Care and Use Committee, conducted in compliance with the Animal Welfare Act, and adhered to the principles stated in the Guide of Care and use of Laboratory Animals published by the US National Institutes of Health.

We assessed the effects of participation with medical intervention versus sedation on efficiency and safety in our colony of cynomolgus macaques (*Macaca fascicularis*) and rhesus macaques (*Macaca mulatta*). Our study focused on a convenience sample of cooperatively trained animals that were sedated for IV access and blood sampling between 22 June 2022 and 5 June 2024 as part of routine health screening. We excluded animals undergoing other procedures, resulting in a total of 7 animals (1 rhesus macaque male and 6 cynomolgus macaques; 2 males and 4 females). We measured total blood sampling time from time of ketamine administration to completion of blood collection and total procedure time/time to return to normal activity from ketamine administration to time of discharge. To compare, we studied 12 cooperatively trained NHPs (4 rhesus macaque males and 8 cynomolgus macaques; 7 males and 1 female) that participated with IV access and blood sampling without sedation for routine health monitoring between 22 May 2023 and 5 June 2024. We measured total blood sampling time from when the animal presented its hind limb to one of several experienced handlers, who had equivalent experience and comparable relationships with animals, until completion of blood collection. We additionally present case studies of 3 additional rhesus macaques, 2 males and 1 female. All animals were purpose-bred and acquired through institutionally approved commercial vendors. During the study period, all animals were housed in same-sex pairs except in rare instances of demonstrated social incompatibility that necessitated single housing. Animals were observed minimally twice daily for general appearance and behavior as a part of routine health monitoring. Water was provided ad libitum, and primate biscuits (2055c or 7195 Envigo Harlan Teklad Nonhuman Primate Diet, or 5048 LabDiet Certified Primate Diet) were provided twice daily based on body weight and supplemented with additional food enrichment consisting of fresh fruits, vegetables, grains, beans, and nuts. Room temperature was maintained at 20–26.7 degrees C, humidity was maintained at 30–70%, and lights were programmed to a 12 h-on, 12 h-off circadian light cycle with 30 min dawn/dusk intervals. Weights were collected at least once a month, veterinary rounds were performed weekly for routine evaluation, and full veterinary exams and bloodwork, including complete blood count and chemistry, were performed at least annually. 

### 2.2. Behavioral Management Program

All animals engaged in a comprehensive behavioral management program that encompassed enrichment activities, social interaction, and structured training sessions. A central component of this program involved all animals participating in an environmental enrichment regimen featuring exposure to novel toys, music, social play, and regular access to spacious play and exercise areas with swimming access. This enrichment plan was designed to provide a rotating variety of enrichment types to provide appetitive, sensory, cognitive, physical, and social enrichment.

Furthermore, to foster increased confidence and positive engagement with routine laboratory and management procedures, and provide an additional cognitive enrichment opportunity, all animals were trained for cooperation with basic husbandry and clinical care tasks, such as shifting, targeting, oral drug administration, and other husbandry-related tasks. Animals underwent training for the cooperative presentation of a hind limb to facilitate the performance of procedures such as sampling, drug and fluid administration, and health monitoring cooperatively in their home environment. This training utilized a mixed-reinforcement program commonly employed at our center, as previously detailed [24,26]. Rhesus macaques completed the cooperative hind limb presentation training in an average of 2.4 h, with an average of 19 sessions over 116 days. Cynomolgus macaques took an average of 3.7 h to complete training, with an average of 26 sessions over 137 days. Both rhesus and cynomolgus macaques had 1–5 training sessions per week relative to schedule demands until completion of training. Average duration of training sessions ranged from 1–12 min and was dependent on both structured training phases and individual animals. Details on the training process and criteria for the satisfactory acquisition of hind limb presentation skills have been previously described [24,26]. Animals trained for cooperative IV sampling or drug administration were implanted with a vascular access port (VAP) that was peripherally placed via the saphenous vein with the tip located in the inferior vena cava [27]. 

### 2.3. IV Access for Blood Sampling and Animal Monitoring

All animals were separated from their social partners for IV access. Complete blood counts (CBCs) and chemistry panels were performed to monitor health. IV access was established using either the VAP or a disposable catheter to collect 1.1 mL of blood. Both groups were evaluated after sampling for side effects.

**Figure 1:**
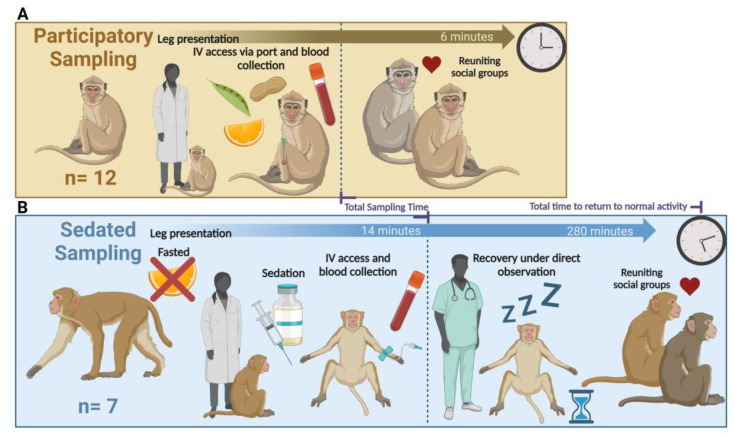
Illustration displaying the process of blood collection from participatory and sedated sampled NHPs. (**A**) Demonstrates the process of cooperative, participatory blood sampling from NHPs with VAPs. (**B**) Demonstrates the process of blood sampling from sedated NHPs. The vertical dotted line marks the completion of sample acquisition during handling and blood collection, indicating the “total sampling time” measured for both participatory and sedated NHPs.

In the participatory cohort, NHPs cooperatively presented their hind limbs with a VAP for IV access and blood sampling in their home environment (Figure 1A). They were immediately re-paired with their social partners following sampling. 

In the sedated cohort, NHPs underwent morning fasting in anticipation of sedated sampling. They cooperatively presented a hind limb for intramuscular administration of ketamine (6–10 mg/kg). After reaching a light plane of sedation, they were briefly removed from their home environment and IV access was established using a 20–22 g butterfly catheter in either the saphenous or cephalic vein for blood sampling. These veins are large peripheral vessels that are easily accessible and can be quickly cannulated under direct visualization. Following blood collection, ketamine-sedated NHPs returned to their home enclosures under direct monitoring. Once they were alert and responsive, demonstrated by the ability to perch and move without ataxia, showed selective or good appetite, and no longer displayed any other adverse effects of sedation, direct monitoring was discontinued and NHPs were re-paired with their social partners (Figure 1B). 

The total sampling time for participatory animals was measured from start of cooperative hind limb presentation to completion of blood collection. For sedated animals, the total sampling time was measured from the time of ketamine administration to the completion of blood collection. Additionally, we compared the total procedure time, defined as time taken to return to normal activity, starting from either the cooperative hind limb presentation (in participatory sampling) or start time of ketamine administration (in sedated sampling). For sedated animals, this included the time required for recovery. All animals were monitored for side effects related to the handling method, such as vomiting, inappetence, shivering, lethargy, and any other behavioral changes from their usual patterns.

### 2.4. Statistical Analysis 

Data were analyzed using non-parametric, two-sided, unpaired Mann–Whitney tests to compare the differences in total sampling time of cooperative, participatory NHPs to total sampling and discharge times of sedated NHPs. The reported results were considered significant if *p* < 0.05 (*), *p* < 0.01 (**), *p* < 0.001 (***), and *p* < 0.0001 (****). Descriptive statistics were used to assess the frequency of side effects during sampling events for participatory and sedated NHPs. Data were analyzed using the statistical software GraphPad Prism version 10.2.3 (Boston, MA, USA). This study was performed as a convenience sample, which was not balanced for sex or species, limiting our ability to definitively evaluate the impact of these variables on outcomes. However, for standardized metrics, such as time taken for procedure, which is not specific to sex or species, these factors are not expected to influence the results.

## 3. Results

### 3.1. Behavioral Management Impact on Intervention Duration

**Figure 2:**
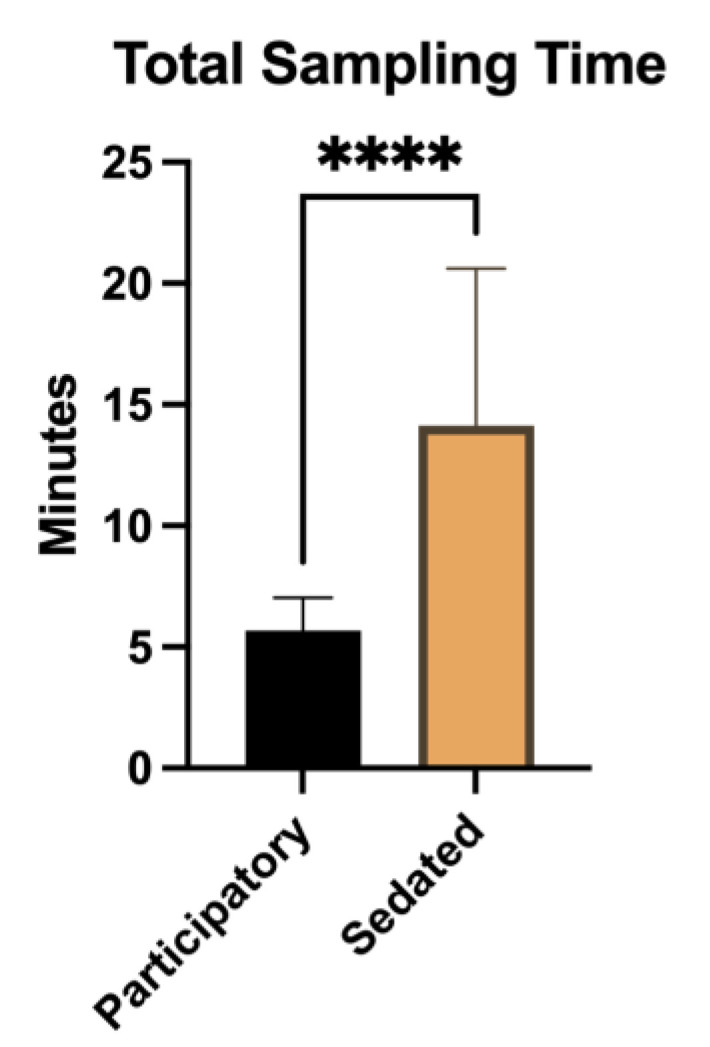
Total sampling time of blood collection for a complete blood count and blood chemistry from participatory sampled macaques compared to sedated sampled macaques. The total sampling time of blood collection from sedated animals was significantly longer than that from participatory animals (*p* < 0.0001). Lines represent standard deviation, **** = *p* < 0.0001.

The total sampling time of the CBC/chemistry blood collection from the participatory NHPs was compared to the total sampling time from the sedated NHPs. The total sampling time was significantly longer for the sedated animals compared to the cooperatively sampled animals (U = 0.500, *p* < 0.0001). The average total sampling time for the participatory animals was 5.7 ± 1.4 min, while the sedated animals had an average total sampling time of 14.1 ± 6.5 min (Figure 2). 

### 3.2. Behavioral Management Impact on Medical Intervention and Recovery (Total Duration)

**Figure 3:**
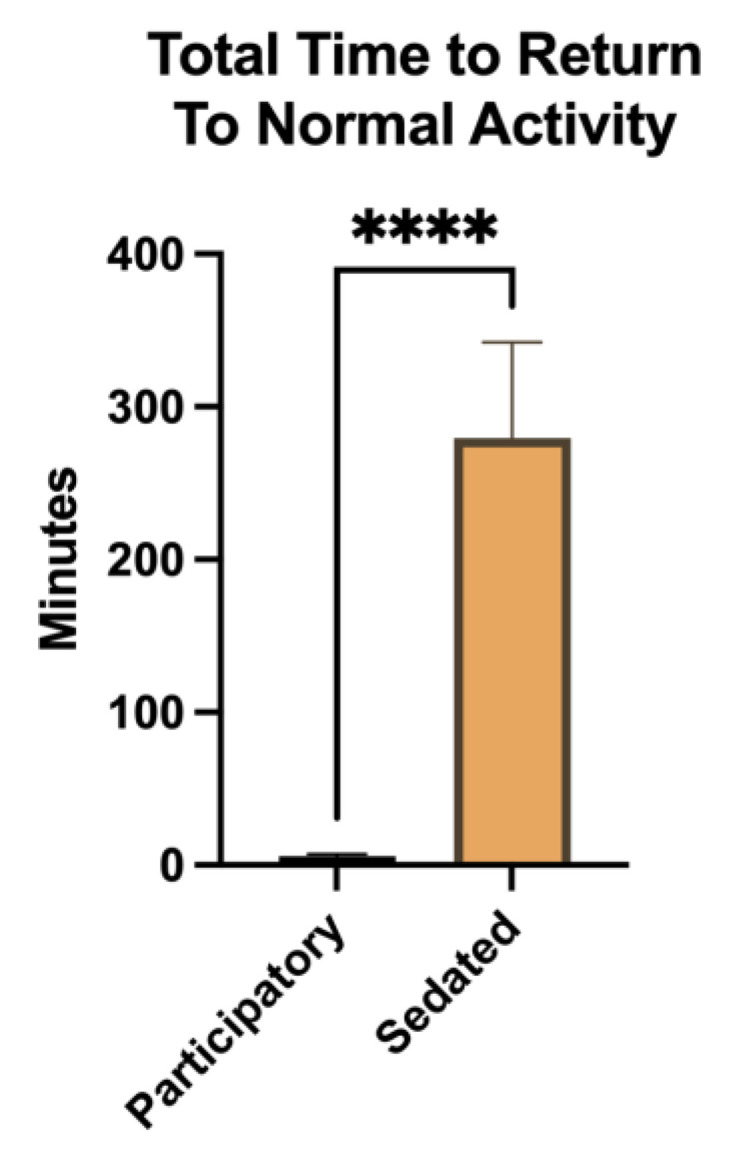
Total sampling time and time to return to normal activity. Blood collection for a complete blood count and blood chemistry compared between participatory and sedated NHPs. The total time of blood collection and return to normal activity for sedated animals was significantly longer than that for participatory sampled animals (*p* < 0.0001). Lines represent standard deviation, **** = *p* < 0.0001.

The total procedure time was compared, recognizing that sedated NHPs require additional monitoring to ensure a safe recovery. The duration was significantly longer for the sedated animals compared to the participatory animals (U = 0, *p* < 0.0001). On average, the sedated animals required 279.6 ± 62.5 min for sampling and full recovery, while the participatory animals, which required no recovery and could be immediately re-paired, took only 5.7 ± 1.4 min for sampling alone (Figure 3).

### 3.3. Side Effects

**Figure 4:**
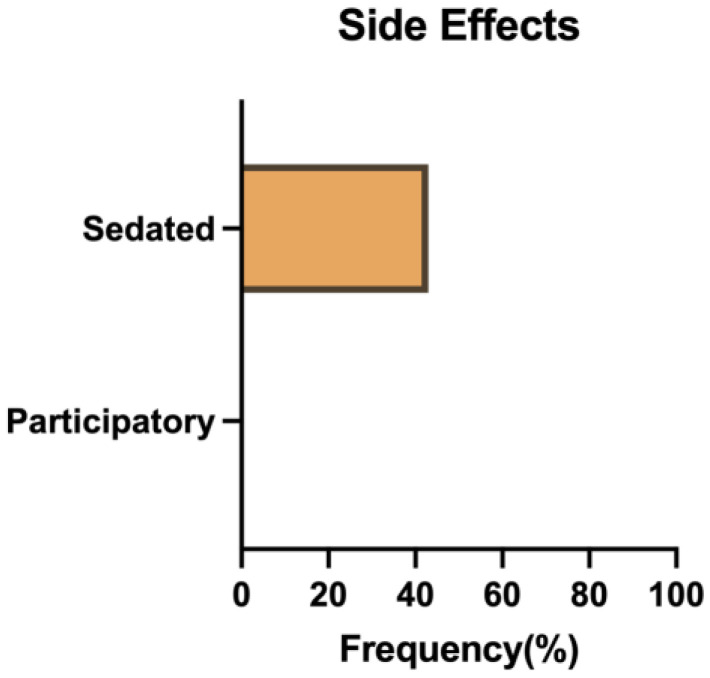
Frequency of side effects related to blood collection of sedated and participatory NHPs.

All animals were monitored for the occurrence of side effects following blood sampling. Side effects were observed in nearly half of the sedated animals (43%), whereas no such issues were noted in the participatory animals. These included two instances of post-sedation vomiting and one instance of reduced appetite (low biscuit consumption) (Figure 4).

### 3.4. Programmatic Impact and Total Animal Burden

**Figure 5:**
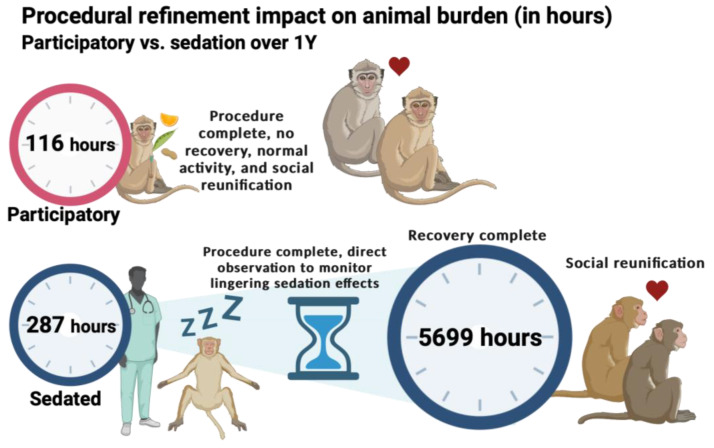
Illustration depicting the difference in burden between animals trained for cooperative IV access compared to those requiring sedation. Blood sampling and drug administration are crucial procedures in NHPs used for translational research applications in our program. In 2023, IV access was performed 1223 times. We used this programmatic scale to extrapolate the broad impact of refined techniques on the program efficiency and animal burden, based on the average times for participatory and sedated IV observed in this study. Based on these data, if animals are involved in their own care, the total time burden for IV access and sample acquisition is 116 h, with no recovery of loss of social opportunity. In contrast, for sedated animals, the total time burden for IV access and sample acquisition is 287 h, which increases to 5699 h when including both sampling and recovery. Our behavioral management program ensures that NHPs are trained to actively participate in their medical care, significantly reducing the time animals are engaged with or affected by medical procedures by nearly 50-fold.

Due to the profound differences in the intervention duration and total procedure duration plus recovery found between the participatory sampled and sedated sampled animals, we estimated the annual programmatic effects that these methodological differences would have as they related to both the occupational time and animal burden. In 2023, IV access was performed 1223 times in NHPs. Based on the average intervention duration calculated above (5.7 ± 1.4 min in the participatory animals, 14.1 ± 6.5 min in the sedated animals), the total time invested in these sampling events is approximately 116 h when NHPs participate in the sampling. In contrast, if these animals had required sedation, the total time investment would have more than doubled to 287 h (Figure 5). Moreover, when factoring in the recovery time, as calculated above (none in the participatory animals, and 279.6 ± 62.5 min in the sedated animals), the burden on the animals increases significantly to approximately 5699 h (Figure 5).

### 3.5. Application of Behavioral Management for Veterinary Care—Case Studies

Here, we present three case studies to illustrate how behavioral management profoundly enhances the implementation of effective and reduced-stress veterinary intervention, care, and monitoring across a spectrum of spontaneously occurring health conditions. 

#### 3.5.1. Case 1—Wound Management 

A 5.2-year-old naive male rhesus macaque weighing 12.3 kg was sedated for initial quarantine veterinary examination, revealing a chronic foot lesion of unknown origin on the plantar surface of the left foot, extending from the distal end of the first digit to the middle of the sole, characterized by multifocal areas of superficial skin ulceration, mild serous discharge, and minimal swelling and erythema. Otherwise, the animal was healthy. A wound culture was obtained, the lesion was flushed with normal saline and cleaned with Hibiclens, and Silvex wound gel was applied. A follow-up veterinary examination was planned, with topical treatment dependent on the next sedation event. Following recovery, the animal exhibited normal behavior, appetite, and activity, but an atypical gait was observed, intermittently characterized by the occasional lifting of the affected foot during ambulation. Environmental factors, systemic disease, vascular disorders, and recent trauma were ruled out as contributing factors. 

While in quarantine, behavioral training for the cooperative presentation of the affected limb (left hind limb) was initiated to facilitate examination and topical treatment administration; within 23 sessions (session length ranged from 1–12 min depending on the phase; the training process details have been previously described [24,26]), the animal had successfully presented the affected foot cooperatively to a familiar handler. This facilitated a routine assessment of the lesion, which would have otherwise been almost impossible to closely visualize without sedation. Similarly, it enabled wound cleaning and the application of topical treatments to prevent infection, promote wound healing, and provide analgesia. On the planned follow-up comprehensive sedated exam, the lesion was markedly improved. The multifocal areas of superficial ulceration transitioned to erosions, with good granulation of the skin and reduced erythema and swelling. Lesions of this nature can pose long-term challenges, especially at sites exposed to continuous friction and pressure. Training enabled the animal to cooperatively participate in the bidaily assessment of this site, which was crucial for promptly recognizing any setbacks and prompt intervention had the status of the lesion worsened. It also facilitated local treatments as directed by the veterinarian based on the site’s condition.

#### 3.5.2. Case 2—Arthritis 

An obese but previously healthy 8.0-year-old male rhesus macaque weighing 16.2 kg was sedated for an annual examination including standard laboratory assessments. The full veterinary physical examination was normal, but bloodwork revealed elevated CRP levels (42 mg/L), suggesting inflammation. Although the animal was clinically normal, approximately 2 weeks later it was sedated for a planned follow-up to reassess the CRP. Following recovery from sedation, the animal displayed an abnormal gait, favoring his left hind leg with intermittent periods of non-weight bearing, toe touching, and the occasional dragging of the left foot. He was otherwise behaving normally and exhibited no other clinical abnormalities. The laboratory workup revealed ongoing inflammation, with a progressive increase in the CRP levels (156 mg/L) and leukocytosis marked by neutrophilia. Considering the animal’s obesity, which increases mechanical stress on joints and promotes inflammation, alongside the observed changes in gait and range of motion without other clinical issues, arthritis with an inflammatory component was suspected.

The veterinary treatment plan prescribed 12.5 mg/kg ibuprofen PO BID for 4 days, alongside the initiation of corticosteroid therapy with methylprednisolone administered intramuscularly once daily. This animal was previously trained to present both hind limbs for examination or drug administration, facilitating cooperation with a 6-week methylprednisolone regimen that started with a dose of 1.6 mg/kg and gradually tapered over the 6-week period. Similarly, the animal participated in hands-on range-of-motion and mobility assessments in his home enclosure, consisting of manual limb manipulation while in hold to assess the ease and degree of flexion and the extension of his affected limb. This also allowed for nonpharmacologic management that included heat and cold therapy. Three weeks into treatment, physical examination revealed mild swelling of the left knee and slightly reduced range of motion. Concurrently, bloodwork indicated a decreased inflammatory state, with a relative reduction in white blood cells, neutrophils, and CRP levels (71.9 mg/L) compared to previous measurements. This suggests a positive response to the therapeutic regimen, indicating improvement in the animal’s condition following initial management. Oral bidaily glucosamine supplementation was initiated 4 weeks after the initial presentation. Despite the abnormal gait persisting throughout the 6-week corticosteroid treatment period, there was gradual improvement noted. Ultimately, the condition completely resolved approximately 12 weeks after the initial presentation. Repeat bloodwork 5 months later showed a continued reduction in CRP levels (22.2 mg/L). This outcome suggests that the combination of corticosteroid therapy, glucosamine supplementation, and manual therapy contributed to the eventual resolution of the animal’s symptoms.

At the time of clinical presentation, this animal had already completed successful training to undergo exams, injections, and physical manipulation by a handler on cue within its home enclosure, thereby minimizing stress due to the animal’s acclimation and counterconditioning to aversive stimuli such as medically necessary injections and physical contact with handlers.

#### 3.5.3. Case 3—Acute and Supportive Care

A previously healthy, naive 3.9-year-old female rhesus macaque weighing 5.28 kg recently released from quarantine presented with acute onset lethargy, trembling, pallor, nausea, and atypical feces characterized by the rapid onset of diarrhea, mucus, and frank blood overnight. This animal completed shift-box training and the early stages of cooperative limb presentation training during quarantine, but she was not fully trained for hands-on examination in her home enclosure. Consequently, she required sedation for examination, during which she was found to be hypothermic (33.3 °C) with severe dehydration. Fluoroscopy, bloodwork, and ultrasound of the abdomen and heart were all unremarkable, suggesting an acute onset of diarrheal illness. Immediate treatment included placement on a forced-air patient-warming device with instant hot packs, and the intravenous administration of 200 mL warmed PlasmaLyte supplemented with 2 g of 50% dextrose solution via infusion pump. Subsequently, an additional 500 mL of PlasmaLyte and 250 mL of 0.9% saline were administered IV, along with 1 mg/kg Ketoprofen administered IM for alleviating potential pain due to dehydration. The veterinary treatment plan prescribed fluoroquinolones, typically effective for shigellosis, salmonellosis, and certain E. coli infections (5 mg/kg enrofloxacin IM once a day for 10 days). It also prescribed 262 mg bismuth subsalicylate once a day for 5 days, and 31.3 mg simethicone PO once a day for 5 days, with highly palatable supplemental fluids offered cageside daily.

Despite the acceptance of oral fluids, mild dehydration persisted. Therefore, the treatment plan was adjusted to include subcutaneous fluid administration as needed, working with the animal to avoid the need for sedation that could complicate oral intake. This animal was undergoing training for cooperative hind limb presentation, becoming accustomed to close contact with handlers being rewarding, and to the use of the squeeze-back panel (SBP) in her home enclosure as a tool to adjust her positioning without restraint. This enabled her to maintain her position at the front of the enclosure in front of the SBP for the subcutaneous administration of 50 mL of 0.9% saline solution using a 22 ga butterfly catheter between the shoulder blades on four separate occasions without being stressed by the proximity of the handlers or the SBP.

Training animals for every possible clinical scenario they might encounter is impractical. Instead, skill acquisition focuses on building trust, practicing procedural handling in a low-stress environment, and reinforcing behaviors with rewards that the animal values. Despite lacking prior training to present for and participate in handling, subcutaneous fluids were successfully administered. The animal remained calm in close proximity to the handlers, without anxiety-provoked escape attempts. Hydration was normalized during the diarrhea treatment, which was fully resolved in 16 days. Even basic training effectively enhances behavioral flexibility and adaptability, as demonstrated by the successful management of an unexpected case of sudden onset diarrhea without adding stress, thereby facilitating rapid recovery.

## 4. Discussion

The aim of this study was to assess how an intentionally designed behavioral management program influences the experience of captive primates during both routine veterinary procedures, such as examinations, blood collection, and drug administration, as well as required care for the management of a range of a variety of spontaneously occurring health conditions. This investigation explores the practical implications of such a program in enhancing veterinary care, promoting animal health and welfare, and improving scientific outcomes. Overall, our findings objectively demonstrate that behavioral training has broad implications for enhancing clinical care. This includes promoting more efficient care, minimizing procedure-related stress and negative effects such as social disruption, reducing the prevalence of side effects, and increasing the feasibility of various treatment options, as well as facilitating comprehensive health monitoring.

In this study, training the animals to participate with IV access took less than 2.5 h in rhesus macaques and less than 4 h in cynomolgus macaques. This timeframe is consistent with our previous findings, where training was completed in a median of 3 h for rhesus macaques and 5.2 h for cynomolgus macaques [24]. This short training period represents a minimal time investment to establish behaviors that improve both efficiency and welfare long term and mitigate the exponential effect on the overall burden to animals and caregivers. IV access was more than twice as efficient when the animals participated with the handlers compared to when sedation was used, completely avoiding the need for recovery time. This speeds up diagnostics to guide treatment and management and reduces the time that animals spend in stressful situations. This is crucial in managing adverse health events or emergencies, where swift decision making and treatment administration can profoundly impact the prognosis and overall health outcomes [28,29]. This is particularly pertinent to consider at the programmatic level, where both the time investment and impact on animals are greatly magnified. Additionally, it has significant financial implications, as reducing the need for sedation and recovery can decrease the use of sedative agents and reduce the total lab personnel time required for routine handling and sampling procedures.

Reducing the total time spent undergoing stressful procedures can significantly impact both health and welfare. Stress can lead to psychological and physiological consequences in both healthy and unhealthy animals, including profound effects on the immune system [30,31,32,33,34,35], which can, in turn, alter responses to disease and therapy. This has critical implications for study outcomes in research animals. In addition to having an average increased total time required for successful sampling and recovery, the sedated animals also had greater within-group variability compared to the participatory animals. Relative to participation, sedation can have more wide-ranging effects across a group of animals and introduce unintended experimental variability. In humans, stress has been shown to have detrimental effects on patient outcomes in a variety of disease conditions [1], both through direct implications on important physiological processes, such as wound healing [2,36], and indirectly through effects on important aspects of health management, like patient compliance [37,38,39]. These consequences extend to animals receiving care, as elevated stress and anxiety can make it difficult, if not impossible, to follow through with an optimal assessment and treatment plan, to the detriment of animal health [40]. By minimizing the duration of these procedures and fostering the development of positive coping skills, behavioral management effectively reduces the intensity and duration of the stress experienced. This approach facilitates easier patient care and mitigates the adverse effects of stress and anxiety on health and welfare.

In this study, it is important to note that even animals in the sedated group had been trained for cooperative limb presentation and were able to participate in their sedation event. As a result, these animals were still able to benefit from reduced stress relative to animals that would otherwise have required physical restraint to facilitate the administration of a sedative agent, as physical restraint has been shown to have significant effects on stress and stress-related parameters [41,42,43]. This cooperation also likely fostered increased overall efficiency in the sedated animals due to the increased ease of sedation. Without this cooperation, the total time differences between the participatory and sedated animals would have been even greater than those presented here.

Cooperative training objectively improves safety for animals exposed to medical intervention, in this case realized in the clinically significant decrease in the side effect prevalence associated with IV access. This approach offers the benefit of enabling animals to swiftly return to their normal, comfortable daily routines, environments, and social interactions, thereby eliminating the recovery period typically associated with sedation or the risk of injury from restraint. Animals participating with handlers required no manipulation of or exit from their enclosures and were able to immediately resume activity with social partners. In contrast, those that underwent sedated sampling required close monitoring, reduced cage space, and separation from social partners until they fully regained consciousness without any signs of ataxia. They also experienced lingering effects on their appetite and activity postrecovery. Over repeated events, this can have a significant effect on welfare and health, as disruption in routine has been shown to further increase stress and anxiety in the healthcare setting [44,45]. Social separation or disruption can have serious consequences, as the increased perception of social isolation has also been shown in humans and NHPs to have detrimental effects on health outcomes, patient satisfaction, and welfare [46,47], which can hinder therapeutic response and patient care. Stress from handling, social separation, or the recovery of conspecifics in close proximity can also disrupt established social dynamics and negatively affect other animals observing these events. In NHPs, social housing status has been linked to changes in stress and immune function [48,49], and its importance is exemplified by welfare regulations regarding social housing status and increased discussions on its implications in biomedical research [50,51]. Without the requirement of sedation, the cooperatively handled animals experienced no procedure-associated side effects, compared to an increased prevalence of vomiting, nausea, and inappetence in the animals that required ketamine-based sedation. This difference aligns with and is likely caused by the documented side effects of ketamine-based sedation [52,53], which can independently compromise welfare and heighten an animal’s stress level. In animals already experiencing health issues, this can further complicate health monitoring by masking intervention-related effects versus symptoms related to their health condition, such as reduced appetite in a normally food-motivated NHP. Minimizing the necessity for sedation and restraint is crucial for expanding treatment options in NHPs with health conditions where sedation would otherwise be unsuitable [54], thereby widening the scope of feasible treatment modalities.

The value and generalizability of training designed to improve the animal experience were exemplified in our case studies, which successfully facilitated efficient and effective veterinary care and monitoring while minimizing additional stress. During training, NHPs acquire skills to interact with handlers through deliberate enrichment opportunities that cultivate strong caregiver–animal bonds. Handlers gain intimate familiarity with both individual and species-typical behaviors. This facilitates easier routine monitoring of their overall condition and enables the detection of subtle changes in their health and behavior. Moreover, training for cooperation allowed for routine, close contact with these animals, which was essential for the daily assessments described. These assessments would have been challenging to conduct accurately without such close interaction and thus promote more precise and comprehensive evaluations of health progression. Similarly, this reduced the intensity and duration of medical treatment for each of these animals, minimizing the need for sedation and its potential consequences. Treatments such as the application of topical ointments, daily IM injections, examination and manipulation, and subcutaneous fluid administration—procedures that conventionally necessitate physical or chemical restraint—were all performed on awake, cooperating animals. This approach minimized the sedation use, social disruption, and stress that could have otherwise prolonged recovery. Ultimately, in each of these cases, behavioral management played a critical role in facilitating important monitoring and veterinary care without disrupting the animal–caregiver relationship that was critical for performing routine, accurate clinical assessments and administering recommended treatments.

Future studies should continue to emphasize the diverse impacts of behavioral management, including its influence on other stress-sensitive health- and outcome-related parameters, such as immune response and direct indices of stress.

## 5. Conclusions

Cooperative handling not only enhances the animal experience during routine medical care but also facilitates complex hands-on treatments crucial for delivering high-quality medical care.

Overall, developing and implementing effective behavioral management programs are essential for improving the clinical care of NHPs and, consequently, their health and welfare. These programs enable animals to actively participate in their care, make choices, and influence outcomes, resulting in more efficient, comprehensive, high-quality, and low-stress care.

## Data Availability

The data presented in this study are available upon request from the corresponding author.
